# Strong convergence theorems for a common zero of a finite family of *H*-accretive operators in Banach space

**DOI:** 10.1186/s40064-016-2646-y

**Published:** 2016-06-30

**Authors:** Huimin He, Sanyang Liu, Rudong Chen

**Affiliations:** School of Mathematics and Statistics, Xidian University, Xi’an, 710071 People’s Republic of China; Department of Mathematics, Tianjin Polytechnic University, Tianjin, 300160 People’s Republic of China

**Keywords:** *H*-accretive operators, Resolvent operator, Iteration algorithms, Strong convergence, 47H06, 47H10

## Abstract

The aim of this paper is to study a finite family of *H*-accretive operators and prove common zero point theorems of them in Banach space. The results presented in this paper extend and improve the corresponding results of Zegeye and Shahzad (Nonlinear Anal 66:1161–1169, 2007), Liu and He (J Math Anal Appl 385:466–476, 2012) and the related results.

## Background

 Let *E* be a real Banach space with norm $\Vert \cdot \Vert$ and Let $E^*$ be its dual space. The value of $x^*\in E^*$ at $x\in E$ will be denoted by $\langle x,x^*\rangle$.

The inclusion problem is finding a solution to

1 $$0\in T(x),$$

where *T* is a set-valued mapping and from *E* to $2^E$.

It was first considered by Rockafellar (1976) by using the proximal point algorithm in a Hilbert space ${\mathscr {H}}$ in 1976. For any initial point $x_0=x\in {\mathscr {H}}$, the proximal point algorithm generates a sequence $\{x_n\}$ in ${\mathscr {H}}$ by the following algorithm

2 $$x_{n+1}=J_{r_n}x_n, \quad n=0,1,2,\ldots ,$$

where $J_{r_n}=(I+r_nT)^{-1}$ and $\{r_n\}\subset (0,\infty )$, *T* is maximal monotone operators.

From then on, the inclusion problem becomes a hot topic and it has been widely studied by many researchers in many ways. The mainly studies focus on the more general algorithms, the more general spaces or the weaker assumption conditions, such as Reich (1979, 1980), Benavides et al. (2003), Xu (2006), Kartsatos (1996), Kamimura and Takahashi (2000), Zhou et al. (2000), Maing (2006), Qin and Su (2007), Ceng et al. (2009), Chen et al. (2009), Song et al. (2010), Jung (2010), Fan et al. (2016) and so on. And their researches mainly contain the maximal monotone operators in Hilbert spaces and the *m*-accretive operators in Banach spaces.

Zegeye and Shahzad (2007) studied a finite family of *m*-accretive mappings and proposed the iterative sequence $\{x_n\}$ is generated as follows:

3 $$x_{n+1}=\alpha _nu+(1-\alpha _n)S_rx_n$$

where $S_r:=a_0I+a_1J_{A_1}+a_2J_{A_2}+\cdots +a_rJ_{A_r}$, with $J_{A_i}:=(I+A_i)^{-1}$ for $0<a_i<1$, $i=1,2,\ldots ,r$, $\sum _{i=0}^r a_i=1$.

And proved the sequence $\{x_n\}$ converges strongly to a common solution of the common zero of the operators $A_i$ for $i=1,2,\ldots ,r$.

Recently, Fang and Huang (2003, 2004) respectively firstly introduced a new class of monotone operators and accretive operators called *H*-monotone operators and *H*-accretive operators, and they discussed some properties of this class of operators.

### **Definition 1**

Let $H:\mathscr {H}\rightarrow \mathscr {H}$ be a single-valued operator and $T:\mathscr {H}\rightarrow 2^{\mathscr {H}}$ be a multivalued operator. *T* is said to be *H*-monotone if *T* is monotone and $(H+\lambda T)(\mathscr {H})=\mathscr {H}$ holds for every $\lambda >0$.

### **Definition 2**

Let $H:E\rightarrow E$ be a single-valued operator and $T:E\rightarrow 2^E$ be a multivalued operator. *T* is said to be *H*-accretive if *T* is accretive and $(H+\lambda T)E=E$ holds for all $\lambda >0$.

### *Remark 1*

The relations between *H*-accretive (monotone) operators and *m*-accretive (maximal monotone) operators are very close, for details, see Liu et al. (2013), Liu and He (2012).

From then, the study of the zero points of *H*-monotone operators in Hilbert space and *H*-accretive operators in Banach space have received much attention, see Peng (2008), Zou and Huang (2008, 2009), Ahmad and Usman (2009), Wang and Ding (2010), Li and Huang (2011), Tang and Wang (2014) and Huang and Noor (2007), Xia and Huang (2007), Peng and Zhu (2007). Especially, Very recently, Liu and He (2013, 2012) studied the strong and weak convergence for the zero points of *H*-monotone operators in Hilbert space and *H*-accretive operators in Banach space respectively.

Motivated mainly by Zegeye and Shahzad (2007) and Liu and He (2012), in this paper, we will study the zero points problem of a common zero of a finite family of *H*-accretive operators and establish some strong convergence theorems of them. These results extend and improve the corresponding results of Zegeye and Shahzad (2007) and Liu and He (2012).

## Preliminaries

Throughout this paper, we adopt the following notation: Let $\{x_n\}$ be a sequence and *u* be a point in a real Banach space with norm $\Vert \cdot \Vert$ and let $E^*$ be its dual space. We use $x_n\rightarrow x$ to denote strong and weak convergence to *x* of the sequence $\{x_n\}$.

A real Banach space *E* is said to be uniformly convex if $\delta (\varepsilon )>0$ for every $\varepsilon >0$, where the modulus $\delta (\varepsilon )$ of convexity of *E* is defined by

4 $$\delta (\varepsilon )=\inf \left\{ 1-\left\| \frac{x+y}{2}\right\| :\Vert x\Vert \le 1,\Vert y\Vert \le 1,\Vert x-y\Vert \ge \varepsilon \right\}$$

for every $\varepsilon$ with $0\le \varepsilon \le 2$. It is well known that if *E* is uniformly convex, then *E* is reflexive and strictly convex (Goebel and Reich 1984)

Let $S\triangleq \{x\in E:\Vert x\Vert =1\}$ be the unit sphere of *E*, we consider the limit

5 $$\lim \limits _{t\rightarrow 0}\frac{\Vert x+th\Vert -\Vert x\Vert }{t}$$

The norm $\Vert \cdot \Vert$ of Banach space *E* is said to be Gâteaux differentiable if the limit (5) exists for each $x,h\in S$. In this case, the Banach space *E* is said to be smooth.

The norm $\Vert \cdot \Vert$ of Banach space *E* is said to be uniformly Gâteaux differentiable if for each $h \in S$ the limit (5) is attained uniformly for *x* in *S*.

The norm $\Vert \cdot \Vert$ of Banach space *E* is said to be Fréchet differentiable if for each $x \in S$ the limit (5) is attained uniformly for *h* in *S*.

The norm $\Vert \cdot \Vert$ of Banach space *E* is said to be uniformly Fréchet differentiable if the limit (5) is attained uniformly for (*x*, *h*) in $S\times S$. In this case, the Banach space *E* is said to be uniformly smooth.

The dual space $E^*$ of *E* is uniformly convex if and only if the norm of *E* is uniformly Fréchet differentiable, then every Banach space with a uniformly convex dual is reflexive and its norm is uniformly Gâteaux differentiable, the converse implication is false. Some related concepts can be found in Day (1993).

Let $H{:}E\rightarrow E$ be a strongly accretive and Lipschtiz continuous operator with constant $\gamma$. Let $T{:}E\rightarrow 2^E$ be an *H*-accretive operator and the resolvent operator $J_{H,\rho }^T{:}E\rightarrow E$ is defined by

6 $$J_{H,\rho }^T(u)=(H+\rho T)^{-1}(u) \quad \forall u\in E.$$

for each $\rho >0$. We can define the following operators which are called *Yosidaapproximation*:

7 $$A_\rho =\frac{1}{\rho }\left(I-H\cdot J_{H,\rho }^T\right) \quad for\,all\,\rho >0.$$

Some elementary properties of $J_{H,\rho }^T$ and $A_\rho$ are given as some lemmas in the following in order to establish our convergence theorems.

### **Lemma 1**

(see Xu 2003) *Let*$\{a_n\}$*be a sequence of non-negative real numbers satisfying the following relation:*

8 $$a_{n+1}\le (1-\gamma _n)a_n+\sigma _n,\quad n\ge 0,$$

*where*$\{\gamma _n\}\subset (0,1)$*for each*$n\ge 0$*satisfy the conditions:*

(i) $$\sum _{n=1}^\infty \gamma _n=\infty$$;
(ii) $$\limsup \nolimits _{n\rightarrow \infty }\frac{\sigma _n}{\gamma _n}\le 0$$ or $$\sum _{n=1}^\infty |\sigma _n|<\infty$$;

*Then*$\{a_n\}$*converges strongly to zero.*

### **Lemma 2**

(Reich 1980) *LetEbe a uniformly smooth Banach space and let*$T:C\rightarrow C$*be a nonexpansive mapping with a fixed point. For each fixed*$u\in C$*and*$t\in (0,1)$, *the unique fixed point*$x_t\in C$*of the contraction*$C\ni x \mapsto tu+(1-t)Tx$*converges strongly as*$t\rightarrow 0$*to a fixed point ofT*. *Define*$Q:C\rightarrow F(T)$ by $Qu=s-\lim \nolimits _{t\rightarrow 0}x_t$. *ThenQis the unique sunny nonexpansive retract fromContoF*(*T*), *that is,Qsatisfies the property*

9 $$\langle u-Qu,J(z-Qu)\rangle \le 0,\quad u\in C,z\in F(T).$$

### **Lemma 3**

(Proposition 4.1 in Liu and He 2012) *Let*$H: E\rightarrow E$*be a strongly accretive and Lipschtiz continuous operator with constant*$\gamma$*and*$T: E\rightarrow 2^E$*be aH*-*accretive operator. Then the following hold:*

(i) $$\Vert J_{H,\rho }^T(x)-J_{H,\rho }^T(y)\Vert \le 1/\gamma \Vert x-y\Vert \quad \forall x,y\in R(H+\rho T);$$
(ii) $$\Vert H\cdot J_{H,\rho }^T(x)-H\cdot J_{H,\rho }^T(y)\Vert \le \Vert x-y\Vert \quad \forall x,y\in E,$$ or $$\Vert J_{H,\rho }^T\cdot H(x)-J_{H,\rho }^T\cdot H(y)\Vert \le \Vert x-y\Vert \quad \forall x,y\in E;$$
(iii) $A_\rho$*is accretive and*
  $$\Vert A_\rho x-A_\rho y\Vert \le \frac{2}{\rho }\Vert x-y\Vert \quad for\,all\,x,y\in R(H+\rho T);$$
(iv) $$A_\rho x\in T J_{H,\rho }^T(x)\quad for\,all\,x\in R(H+\rho T).$$

### **Lemma 4**

(Proposition 4.2 in Liu and He 2012) $u\in T^{-1}0$*if and only ifusatisfies the relation*

10 $$u=J_{H,\rho }^T(H(u))$$

*where*$\rho >0$*is a constant and*$J_{H,\rho }^T$*is the resolvent operator defined by* (6).

### **Lemma 5**

(see Petryshyn 1970) *LetEbe a real Banach space. Then for all*$x,y\in E$, $\forall j(x+y)\in J(x+y)$,

11 $$\Vert x+y\Vert ^2\le \Vert x\Vert ^2+2\langle y,j(x+y)\rangle .$$

## Main results

### **Proposition 1**

*LetEbe a strictly convex Banach space,*$H{:}E \rightarrow E$*be a strongly accretive and Lipschtiz continuous operator with constants*$\gamma$. *Let*$T_i{:}E\rightarrow 2^E,i=1,2,\ldots ,r$*be a family ofH*-*accretive operators with*$\cap _{i=1}^rN(T_i)\ne \emptyset$. *Let*$a_0,a_1,a_2,\ldots ,a_r$*be real numbers in* (0, 1) *such that*$\sum _{i=0}^r a_i=1$*and let*$S_r:=a_0I+a_1J_{H,\rho }^{T_1}H+a_2J_{H,\rho }^{T_2}H+\cdots +a_rJ_{H,\rho }^{T_r}H$, *where*$J_{H,\rho }^T=(H+\rho T)^{-1}$. *Then*$S_r$*is nonexpansive and*$F(S_r)=\cap _{i=1}^rN(T_i)$.

### *Proof*

Since every $T_i$ is *H*-accretive for $i=1,2,\ldots ,r$, then $J_{H,\rho }^{T_i}H$ is well defined and it is a nonexpansive mapping from Lemma 4, and we can also get that $F(J_{H,\rho }^{T_i}H)=N(T_i)$.

Hence, it is easy to obtain that

$$\mathop \cap\limits_{{i = 1}}^{r} {N(T_{i} )} =\mathop \cap\limits_{{i = 1}}^{r} {F\left(J_{{H,\rho }}^{{T_{i} }} H\right) \subseteq F(S_{r} )}$$

and $S_r$ is nonexpansive.

Next, we prove that $F(S_r)\subseteq \cap _{i=1}^rF(J_{H,\rho }^{T_i}H)$.

Let $z\in F(S_r)$, $w\in \cap _{i=1}^rF(J_{H,\rho }^{T_i}H)$, we have

12 $$\begin{aligned} z-w &= a_0z+a_1J_{H,\rho }^{T_1}Hz+a_2J_{H,\rho }^{T_2}Hz+\cdots +a_rJ_{H,\rho }^{T_r}Hz-w\nonumber \\&= a_0(z-w)+a_1(J_{H,\rho }^{T_1}Hz-w)+a_2(J_{H,\rho }^{T_2}Hz-w)+\cdots +a_r(J_{H,\rho }^{T_r}Hz-w). \end{aligned}$$

The above equality can be also written as follows:

$$z-w=\frac{a_1}{\sum _{i=1}^r a_i}(J_{H,\rho }^{T_1}Hz-w)+\cdots +\frac{a_r}{\sum _{i=1}^r a_i}(J_{H,\rho }^{T_r}Hz-w)$$

so

13 $$\Vert z-w\Vert =\Vert \frac{a_1}{\sum _{i=1}^r a_i}(J_{H,\rho }^{T_1}Hz-w)+\cdots +\frac{a_r}{\sum _{i=1}^r a_i}(J_{H,\rho }^{T_r}Hz-w)\Vert$$

From (12), we also have

14 $$\begin{aligned} \Vert z-w\Vert &= \Vert a_0(z-w)+a_1(J_{H,\rho }^{T_1}Hz-w)+a_2(J_{H,\rho }^{T_2}Hz-w)+\cdots +a_r(J_{H,\rho }^{T_r}Hz-w)\Vert \nonumber \\&\le a_0\Vert z-w\Vert +a_1\Vert J_{H,\rho }^{T_1}Hz-w\Vert +a_2\Vert J_{H,\rho }^{T_2}Hz-w\Vert +\cdots +a_r\Vert J_{H,\rho }^{T_r}Hz-w\Vert \nonumber \\&\le \sum _{i=0}^{r}a_i\Vert z-w\Vert \nonumber \\&= \Vert z-w\Vert . \end{aligned}$$

From (14), we get

$$\begin{aligned} \Vert z-w\Vert &= \sum _{i=0}^{r}a_i\Vert z-w\Vert \\&= \sum _{i=0}^{r-1}a_i\Vert z-w\Vert +a_r\Vert J_{H,\rho }^{T_r}Hz-w\Vert \\&= (1-a_r)\Vert z-w\Vert +a_r\Vert J_{H,\rho }^{T_r}Hz-w\Vert . \end{aligned}$$

Hence,

$$\Vert z-w\Vert =\Vert J_{H,\rho }^{T_r}Hz-w\Vert .$$

Similarly, we can get

15 $$\Vert z-w\Vert =\Vert J_{H,\rho }^{T_1}Hz-w\Vert =\Vert J_{H,\rho }^{T_2}Hz-w\Vert =\cdots =\Vert J_{H,\rho }^{T_r}Hz-w\Vert .$$

From the strict convexity of *E*, (13) and (15), we know that

$$z-w=J_{H,\rho }^{T_1}Hz-w=J_{H,\rho }^{T_2}Hz-w=\cdots =J_{H,\rho }^{T_r}Hz-w.$$

Therefore,

$$J_{H,\rho }^{T_i}Hz=z, \quad for\ \ i=1,2,\ldots ,r.$$

Namely,

$$z\in \mathop\cap\limits _{i=1}^rF(J_{H,\rho }^{T_i}H)$$

The proof is completed. $\square$

### **Theorem 1**

*LetEbe a strictly convex and real uniformly smooth Banach space which has a uniformly G*$\hat{a}$*teaux differentiable norm,*$H{:}E \rightarrow E$*be a strongly accretive and Lipschtiz continuous operator with constants*$\gamma$. *Let*$T_i{:}E\rightarrow 2^E,i=1,2,\ldots ,r$*be a family ofH*-*accretive operators with*$\cap _{i=1}^rN(T_i)\ne \emptyset$, *For given*$u,x_0\in E$, *let*$\{x_n\}$*be generated by the algorithm*

16 $$x_{n+1}=\alpha _n u+(1-\alpha _n)S_rx_n,\quad n\ge 0.$$

*where*$S_r:=a_0I+a_1J_{H,\rho }^{T_1}H+a_2J_{H,\rho }^{T_2}H+\cdots +a_rJ_{H,\rho }^{T_r}H$, *with*$J_{H,\rho }^{T_i}=(H+\rho {T_i})^{-1}$*for*$0<a_i<1,\,i=1,2,\ldots ,r,\,\sum _{i=0}^r a_i=1$, *where*$\forall \rho \in (0,\infty )$*and*$\{\alpha _n\}\subset [0,1]$*satisfy the following conditions:*

(i) $$\lim \nolimits _{n\rightarrow \infty }\alpha _n=0$$,
(ii) $$\sum _{n=0}^\infty \alpha _n=\infty$$,
(iii) $$\sum _{n=0}^\infty |\alpha _n-\alpha _{n-1}|<\infty$$ or $$\lim \nolimits _{n\rightarrow \infty }\frac{|\alpha _n-\alpha _{n-1}|}{\alpha _n}=0$$,

*Then*$\{x_n\}$*converges strongly to a common solution of the equations*$T_ix=0$ for $i=1,2,\ldots ,r$.

### *Proof*

First, we show that $\{x_n\}$ is bounded.

By the Proposition 1, we have that $F(S_r)=\cap _{i=1}^rN(T_i)\ne \emptyset$. Then, take a point $x^*\in F(S_r)$, we get

$$\begin{aligned} \Vert x_{n+1}-x^*\Vert &\le \Vert \alpha _n (u-x^*)+(1-\alpha _n)(S_rx_n-x^*)\Vert \\&\le \alpha _n \Vert u-x^*\Vert +(1-\alpha _n)\Vert x_n-x^*\Vert . \end{aligned}$$

By induction we obtain that

$$\Vert x_{n}-x^*\Vert \le \max \{\Vert u-x^*\Vert ,\Vert x_0-x^*\Vert \}, \quad for \, n=0,1,2\ldots$$

Hence, $\{x_n\}$ is bounded, and so is $\{S_rx_n\}$.

Second, we will show that $\Vert x_{n+1}-x_n\Vert \rightarrow 0$.

From (16) we can get that

$$\begin{aligned} \Vert x_{n+1}-x_n\Vert &= \Vert \alpha _n u+(1-\alpha _n)S_rx_n-\alpha _{n-1} u-(1-\alpha _{n-1})S_rx_{n-1}\Vert \\&= \Vert (\alpha _n-\alpha _{n-1})u+ (1-\alpha _n)S_rx_n-(1-\alpha _{n-1})S_rx_{n-1}\Vert \\ &= \Vert (\alpha _n-\alpha _{n-1})u+ (1-\alpha _n)S_rx_n-(1-\alpha _n)S_rx_{n-1}\\&\quad +\, (1-\alpha _n)S_rx_{n-1}-(1-\alpha _{n-1})S_rx_{n-1}\Vert \\ &= \Vert (\alpha _n-\alpha _{n-1})(u-S_rx_{n-1})+ (1-\alpha _n)(S_rx_n-S_rx_{n-1})\Vert \\&\le (1-\alpha _n)\Vert S_rx_n-S_rx_{n-1}\Vert +|\alpha _n-\alpha _{n-1}|\Vert u-S_rx_{n-1}\Vert \\&\le (1-\alpha _n)\Vert x_n-x_{n-1}\Vert +|\alpha _n-\alpha _{n-1}|M \end{aligned}$$

where $M=\sup \{\Vert u-S_rx_{n-1}\Vert ,\, n=0,1,2\ldots \}$ for $\{S_rx_n\}$ is bounded. By applying the Lemma 1 and condition (iii), we assert that

$$\Vert x_{n+1}-x_n\Vert \rightarrow 0,$$

as $$n\rightarrow \infty$$.

Then, we have

$$\Vert x_n-S_rx_n\Vert \le \Vert x_n-x_{n+1}\Vert +\Vert x_{n+1}-S_rx_n\Vert ,$$

and so that

17 $$\Vert x_n-S_rx_n\Vert \rightarrow 0,$$

as $$n\rightarrow \infty$$.

Based on the Lemma 2, there exists the sunny nonexpansive retract *Q* from *E* onto the common zeros point set of $T_i$ ($\cap _{i=1}^rN(T_i), \,i=1,2,\ldots r$) and it is unique, that is to say for $t\in (0,1)$,

$$Qu=s-\lim \limits _{t\rightarrow 0}z_t, \quad u\in E,$$

and $z_t$ satisfies the following equation

$$z_t=tu+(1-t)S_rz_t,$$

where $u\in E$ is arbitrarily taken for all $r>0$.

Applying the Lemma 5, we obtain that

$$\begin{aligned} \Vert z_t-x_n\Vert ^2 &= \Vert t(u-x_n)+(1-t)(S_rz_t-x_n)\Vert ^2\\&\le (1-t)^2\Vert S_rz_t-x_n\Vert ^2+2t\langle u-x_n,j(z_t-x_n)\rangle \\&\le (1-t)^2(\Vert S_rz_t-S_rx_n\Vert +\Vert S_rx_n-x_n\Vert )^2+2t(\Vert z_t-x_n\Vert ^2\langle u-x_n,j(z_t-x_n)\rangle )\\&\le (1+t^2)\Vert z_t-x_n\Vert ^2+\Vert S_rx_n-x_n\Vert (2\Vert z_t-x_n\Vert +\Vert S_rx_n-x_n\Vert )\\&\quad +\,2t\langle u-x_n,j(z_t-x_n)\rangle . \end{aligned}$$

Then, we have

$$\langle u-x_n,j(z_t-x_n)\rangle \le \frac{t}{2}\Vert z_t-x_n\Vert ^2+\frac{\Vert S_rx_n-x_n\Vert }{2t}(2\Vert z_t-x_n\Vert +\Vert S_rx_n-x_n\Vert ).$$

Since $\Vert S_rx_n-x_n\Vert \rightarrow 0$ as $n\rightarrow 0$ by (17).

Let $n\rightarrow \infty$, we obtain that

18 $$\limsup \limits _{n\rightarrow \infty }\langle u-x_n,j(z_t-x_n)\rangle \le \frac{t}{2}M,$$

where *M* is a constant such that $\Vert z_t-x_n\Vert ^2\le M$ for all $t\in (0,1)$ and $n=1,2,\ldots$.

Since $z_t\rightarrow Qu$ as $t\rightarrow \infty$ and the duality mapping *j* is norm-to weak$^*$ uniformly continuous on bounded subsets of *E*. Let $t\rightarrow 0$ in (18), we have that

19 $$\limsup \limits _{n\rightarrow \infty }\langle u-Qu,j(x_{n+1}-Qu)\rangle \le 0.$$

Finally, we will show $x_n\rightarrow Qu$. Applying Lemma 5 to get,

20 $$\begin{aligned} \Vert x_{n+1}-Qu\Vert ^2 &= \Vert (1-\alpha _n)(y_n-Qu)+\alpha _n(u-Qu)\Vert ^2\nonumber \\ &\le \Vert (1-\alpha _n)(y_n-Qu)\Vert ^2+2\alpha _n\langle u-Qu,j(x_{n+1}-Qu)\rangle \nonumber \\&\le (1-\alpha _n)(\Vert J_{H,r_{n}}^TH(x_{n})-Qu\Vert +\Vert y_n-J_{H,r_{n}}^TH(x_{n})\Vert )^2\nonumber \\&\quad +\,2\alpha _n\langle u-Qu,j(x_{n+1}-Qu)\rangle \nonumber \\&\le (1-\alpha _n)(\Vert x_n-Qu\Vert +\delta _n)^2+2\alpha _n\langle u-Qu,j(x_{n+1}-Qu)\rangle \nonumber \\&\le (1-\alpha _n)\Vert x_n-Qu\Vert ^2+2\alpha _n\langle u-Qu,j(x_{n+1}-Qu)\rangle +M\delta _n, \end{aligned}$$

where $M>0$ is some constant such that $2(1-\alpha _n)\Vert x_n-Qu\Vert +\delta _n\le M$. An application of Lemma 1 yields that $\Vert x_n-Qu\Vert \rightarrow 0$

This completes the proof. $\square$

### *Remark 2*

If we take $r=1$, $a_0=0,a_1=1$ in Theorem 1, we can get Theorem 4.1 in Liu and He (2012).

### *Remark 3*

If we suppose $T_i$ (i = 1,2,...,r) is *m*-accretive in Theorem 1, we can get Theorem 3.3 in Zegeye and Shahzad (2007).

## Conclusions

In this paper, we considered the strong convergence for a common zero of a finite family of *H*-accretive operators in Banach space using the Halpern iterative algorithm (16). The main results presented in this paper extend and improve the corresponding results of Zegeye and Shahzad (2007) and Liu and He (2012) and the related results.
